# Transcriptome architecture reveals genetic networks of bolting regulation in spinach

**DOI:** 10.1186/s12870-021-02956-0

**Published:** 2021-04-14

**Authors:** Reza Abolghasemi, Maryam Haghighi, Nematollah Etemadi, Shui Wang, Aboozar Soorni

**Affiliations:** 1 Department of Horticulture, College of Agriculture, Isfahan University of Technology, Isfahan, Iran; 2 College of Life Sciences, Shanghai Normal University, Shanghai, China; 3 Department of Biotechnology, College of Agriculture, Isfahan University of Technology, Isfahan, Iran

**Keywords:** Bolting, Flowering time, Spinach, RNA-Seq

## Abstract

**Background:**

Bolting refers to the early flowering stem production on agricultural and horticultural crops before harvesting. Indeed, bolting is an event induced by the coordinated effects of various environmental factors and endogenous genetic components, which cause a large reduction in the quality and productivity of vegetable crops like spinach. However, little is known about the signaling pathways and molecular functions involved in bolting mechanisms in spinach. The genetic information regarding the transition from vegetative growth to the reproductive stage in spinach would represent an advantage to regulate bolting time and improvement of resistant cultivars to minimize performance loss.

**Results:**

To investigate the key genes and their genetic networks controlling spinach bolting, we performed RNA-seq analysis on early bolting accession Kashan and late-bolting accession Viroflay at both vegetative and reproductive stages and found a significant number of differentially expressed genes (DEGs) ranging from 195 to 1230 in different comparisons. These genes were mainly associated with the signaling pathways of vernalization, photoperiod/circadian clock, gibberellin, autonomous, and aging pathways. Gene ontology analysis uncovered terms associated with carbohydrate metabolism, and detailed analysis of expression patterns for genes of Fructose-1, 6-bisphosphate aldolase, TREHALOSE-6-PHOSPHATE SYNTHASE 1, FLOWERING PROMOTING FACTOR 1, EARLY FLOWERING, GIGANTEA, and MADS-box proteins revealed their potential roles in the initiating or delaying of bolting.

**Conclusion:**

This study is the first report on identifying bolting and flowering-related genes based on transcriptome sequencing in spinach, which provides insight into bolting control and can be useful for molecular breeding programs and further study in the regulation of the genetic mechanisms related to bolting in other vegetable crops.

**Supplementary Information:**

The online version contains supplementary material available at 10.1186/s12870-021-02956-0.

## Background

Spinach (*Spinacia oleracea* L.) from the Amaranthaceae family is an annual, dioecious, and cold-tolerant leafy vegetable plant with diverse nutrients and health-promoting compounds such as fiber, vitamins, iron, and antioxidant activities [1, 2]. This plant is cultivated worldwide and is becoming one of the most important economic vegetable crops with an estimated annual production of ~ 26 million tonnes [3]. Indeed, spinach’s economic value is significantly affected by the yield performance, which is greatly affected by environmental factors, including light, temperature, water, humidity, and nutrition [4, 5]. Bolting or premature flowering, a critical event due to the coordinated effects of various environmental factors and endogenous genetic components [6, 7], is known as one of the most important factors which can cause a large reduction in quality, productivity, and serious economic loss in spinach crop, particularly in the spring [8–10]. According to the time of bolting, spinach cultivars are divided into three categories: “early-bolting” cultivars, the floral stem appears earlier than 60 d after planting, “intermediate-bolting” cultivars, bolting time is between 60 and 70 d, and “late-bolting” cultivars, bolting occurs after the 70 d. Late-bolting spinach varieties can cut multiple times during the growing season and increases the overall yield because they are not sensitive to photoperiod [9]. Hence, the use of late-bolting cultivars and regulation of bolting time are the most successful ways to limit the effect of bolting on spinach productivity. Moreover, research on the signaling pathways and molecular functions of flowering-related genes can enable researchers to regulate bolting.

In recent years, many studies have revealed flowering-related signaling pathways and regulatory networks in model plants [11–15], but few studies have focused on spinach. Studies on Arabidopsis [15], radish [16], carrot [17], and lettuce [18] have discovered substantial information regarding the influence of photoperiod, aging, vernalization, endogenous hormones (especially gibberellins), and signal cascades on bolting. These signals regulate the most important flowering integrators, including SUPPRESSOR OF OVEREXPRESSION OF CO1 (SOC1), FLOWERING LOCUS T (FT), and LEAFY (LFY) that determine the eventual flowering time. The key gene in the photoperiod pathway is CONSTANS (CO), a transcription factor that positively activates FT [19, 20]. In the vernalization pathway, FLOWERING LOCUS C (FLC) is known as a key gene that suppresses flowering through inhibiting SOC1, FT, and LFY genes [21, 22]. SQUAMOSA PROMOTER BINDING-LIKE PROTEIN 1 (SPL1), SPL2, SPL3, SPL9, SPL13, and SPL15 are the most relevant candidate genes related to the aging pathway [23]. Based on both physiological and genetic studies, a decrease in gibberellin content affects flower formation by restricting internode elongation. LFY is one of the main target genes in the gibberellins pathway [24].

Most of the information has been uncovered over the past few years by using Next-generation sequencing (NGS) technologies. Nowadays, RNA-Seq combined with Digital gene expression (DGE) profiling is a powerful strategy for the global discovery of functional genes and expression analysis under certain conditions in many plant species. For example, RNA-seq has been applied for the detection of genes linked with the flowering process in *Arabidopsis thaliana* [25], *Raphanus sativus* [16], *Eichhornia paniculata* [26], and *Lagerstroemia indica* [27], and *Lactuca sativa* [18].

Previous studies have reported different genes, pathways, mechanisms, and networks have complex roles in flowering induction that are varied in different plant species under different circumstances [15–18]. Although the spinach reference genome has been sequenced [28] by using NGS technologies and bioinformatics approaches, and transcriptome studies are currently in progress, little is known about the genes and genetic networks involved in flowering and bolting mechanisms from vegetative growth to the reproductive stage in spinach. Thus, we performed transcriptome and qPCR analyses to reveal key genes associated with spinach bolting. In this regard, two leafy spinach accessions with different bolting resistance potential, including “Kashan” (early bolting accession), and “Viroflay” (late-bolting accession), were chosen as materials for high-throughput RNA sequencing (RNA-seq) and analyze global transcripts level changes in two vegetative to reproductive stages. Our findings will contribute to identify genes and molecular mechanisms regulating bolting, which could help us to better understand the bolting mechanisms in spinach and can be useful for molecular breeding programs and further study in the regulation of the genetic mechanisms related to bolting in other vegetable crops.

## Results

### Sequencing data analysis

**Fig. 1:**
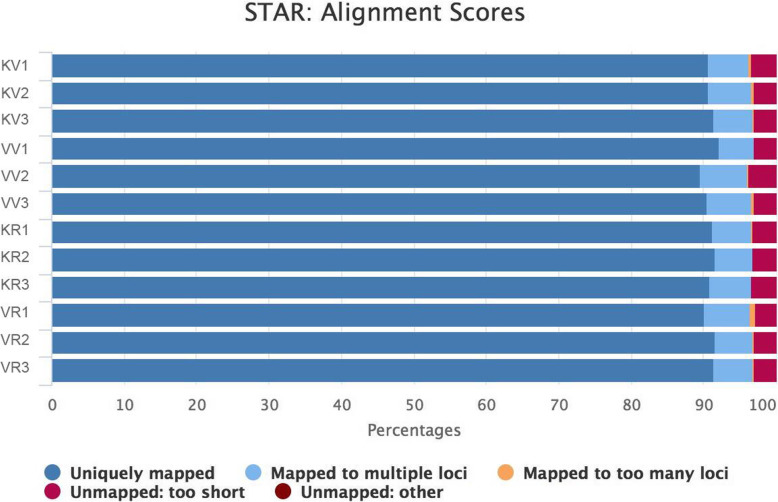
Quality control of alignment, a bar plot with STAR alignment rates of 12 spinach transcriptome samples. KV, KR, VV, and VR represent Kashan-vegetative, Kashan-reproductive, Viroflay-vegetative, and Viroflay-reproductive, respectively

A total of 559.064 million raw reads were generated from 12 cDNA libraries constructed with leaf samples of two accessions Kashan and Viroflay at vegetative and reproductive stages. The average output of each sample was 46.588 million. Of these reads, an average of 43.269 million clean reads with a Q30 ratio of more than 92.86% was retained from each library after removing low-quality reads and adaptor sequences (Table S1). After the quality check, approximately a range of 97.80–98.30% high-quality sequencing reads of each sample were individually mapped to spinach genome assembly using STAR (Fig. 1, Table S2).

### Differential gene expression profiling

**Fig. 2:**
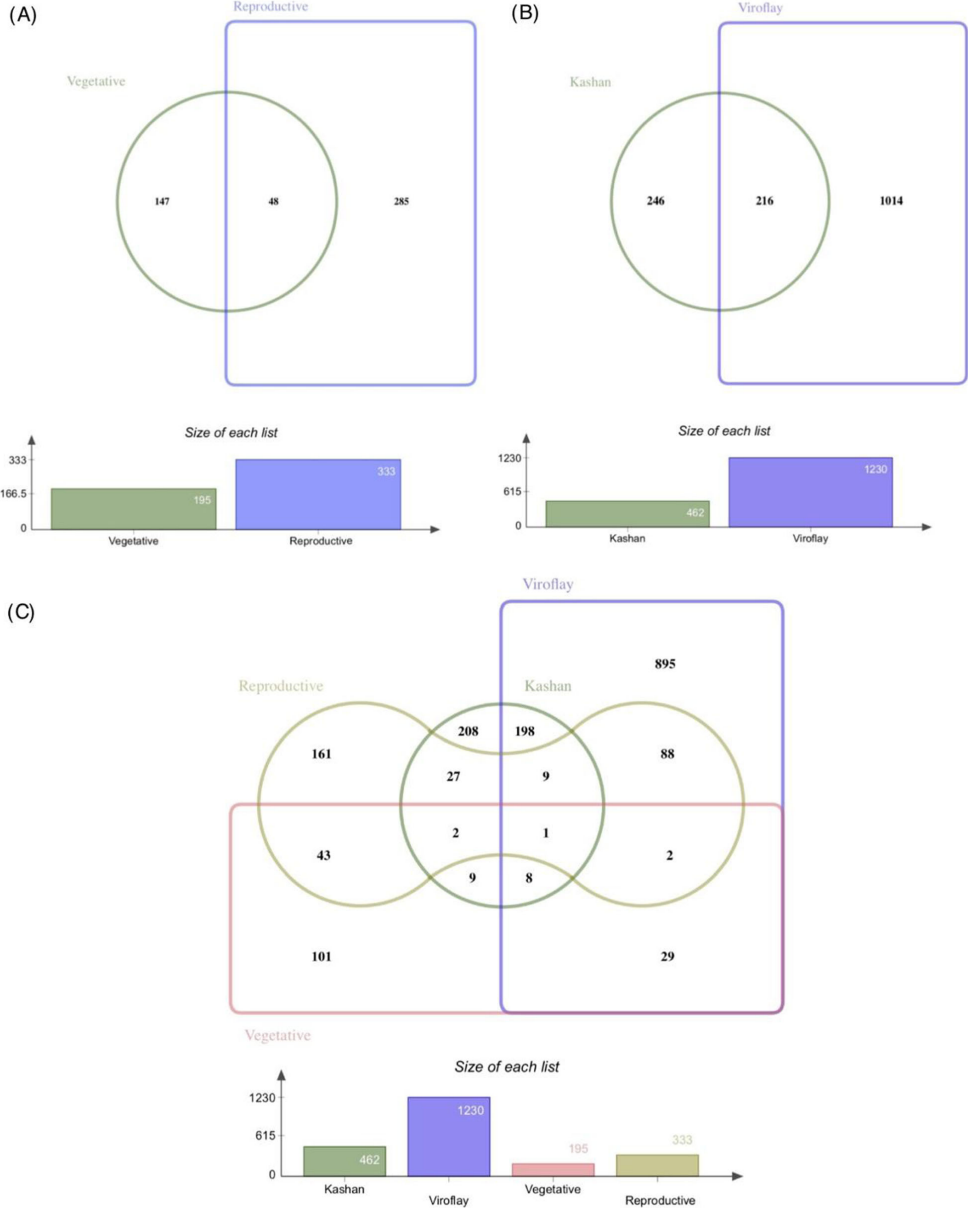
Venn diagram showing the distribution of unique and common DEGs among comparisons. The number of DEGs commonly shared between stages in the accessions comparison (**a**), between accessions under stages comparison (**b**) and both stages and accessions comparison (**c**)

To discover the genetic differences between early and late-bolting accessions, DEGs were identified from pairwise comparisons of both stages and accessions (Fig. 2). Developmental stages comparison in each accession revealed a total of 462 and 1230 genes as significant DEGs in Kashan and Viroflay (Fig. 2b), respectively, with a strong DEG signal for upregulated genes in the reproductive stage. The accessions’ comparison in each stage exhibited that the number of DEGs at the reproductive stage (333 genes) was significantly more than that at the vegetative stage (195 genes) (Fig. 2a). Interestingly, at the vegetative stage, more upregulated genes were associated with accession Kashan, whereas at the reproductive stage, more upregulated genes were found in Viroflay. Furthermore, there were 216 DEGs commonly shared between accessions under stages comparison, while 48 DEGs were found to be common between stages in the accessions comparison (Fig. 2a). To obtain a more comprehensive view, the distribution of unique and common DEGs identified in each pairwise comparisons of stages and accessions was shown in Fig. 2c. Additionally, to characterize the subgenome and chromosome distribution of DEGs, the location data and log2 fold change of DEGs in each comparison were identified and plotted on 6 chromosomes of spinach (Fig. S1-S2). It was found that chromosome numbers 1 and 2 have the highest number of DEGs in vegetative stages comparison, similar to stages comparison in accessions Kashan and Viroflay, while DEGs related to reproductive stages comparison showed a more balanced dispersion across all the 6 chromosomes. A file containing the list of DEGs (DataS1) is provided in the supplementary material.

### Gene classification of predicted-DEGs

To address the product properties and functional classification of the DEGs, we adopted Gene Ontology (GO) terms for all DEGs identified in pairwise comparisons of stages and accessions using Gene Classification tools located in SpinachBase [29].

**Fig. 3:**
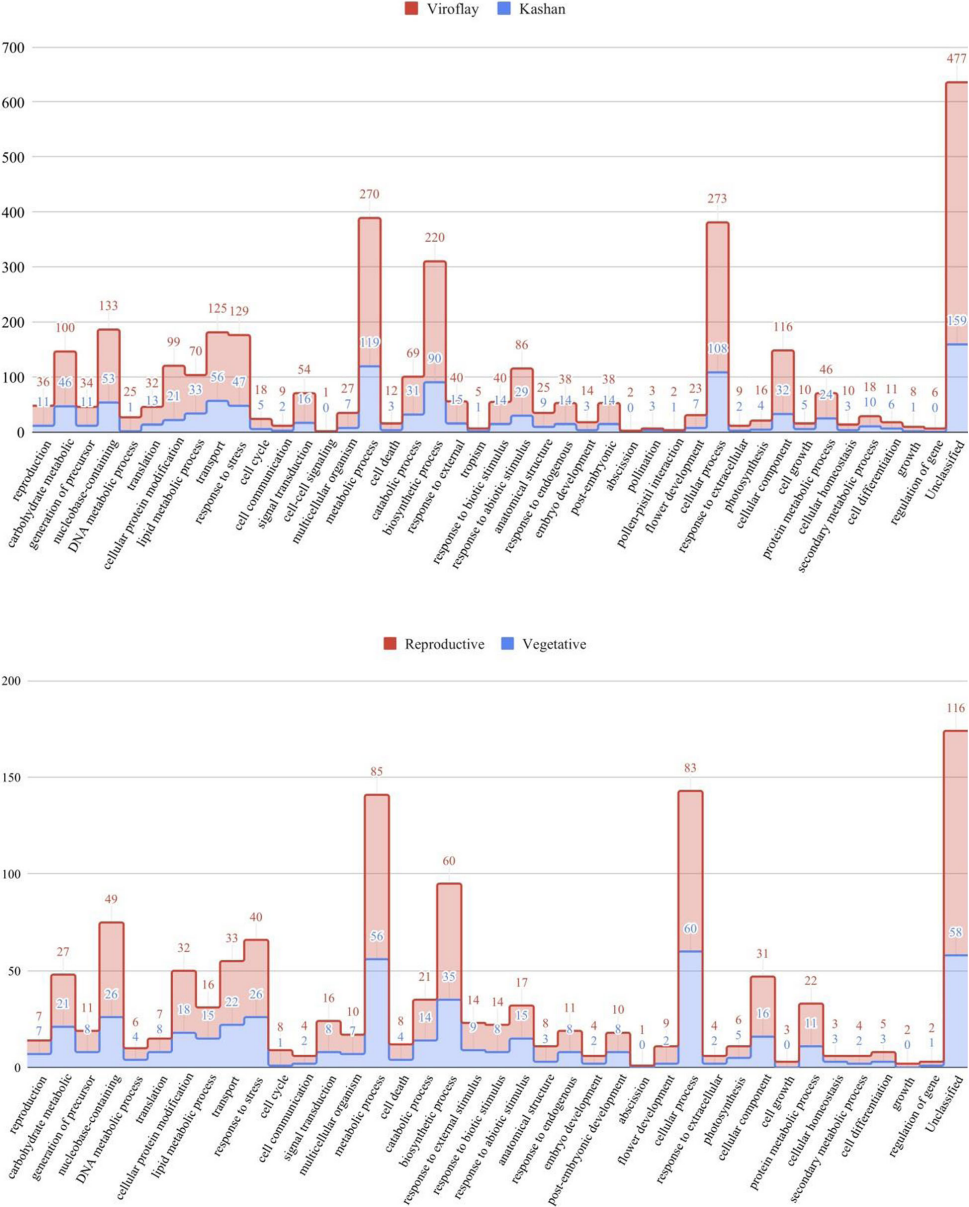
Gene classification of identified DEGs. The histogram shows the classification of DEGs under the biological process category for all pairwise comparisons

With respect to the stages comparison in accession Kashan, of the 462 DEGs, 303, 325, and 200 were successfully annotated with GO assignments in the three main categories, including biological process (BP, Fig. 3), molecular function (MF, Fig. S3), and cellular component (CC, Fig. S4), respectively. The counterpart numbers of DEGs derived from the Viroflay were 753, 812, and 482 genes, respectively. With respect to the accessions comparison in the vegetative stage, 137, 139, 80 genes were involved in different GO terms of BP, MF, and CC categories, respectively. According to the details of the GO analysis of 333 DEGs that were for the reproductive stage, BP was the dominant category with 275 genes, followed by MF with 220 and CC with 125 genes.

In order to obtain a deeper and better understanding, further analysis included only the GO terms associated with biological processes. Enrichment results revealed that similar functional subcategories, including ‘metabolic processes’, ‘cellular processes’, and ‘biosynthetic processes’ were dominant under the biological processes in all comparisons. Besides that, a more number of DEGs were classified into reproduction (GO:0000003) and flower development (GO:0009908) subclasses in stages comparison of each accession versus accessions comparison in each stage, which is related to the developmental activities taking place during flowering. Additionally, several DEGs were annotated with GO terms related to embryo development (GO:0009790), post-embryonic development (GO: 0009791), pollination (GO: 0009856). These processes are well known to be involved in the transition from the vegetative stage to the reproductive stage.

Moreover, to summarize and characterize DEGs putatively implicated in bolting regulatory networks, the most popular genes were identified and classified in various flowering pathways, including vernalization, photoperiod/circadian clock, GA, autonomous, and aging pathways. In the comparison of vegetative and reproductive stages of each accession, the searching results indicated that 18 and 20 functional genes such as CONSTANS-LIKE 1 (COL1), EARLY FLOWERING 3 (ELF3), FLOWERING LOCUS T (FT), AGAMOUS-LIKE (AGL), and GIGANTEA (GI) were identified and implicated in photoperiod pathway of accessions Kashan and Viroflay, respectively. Besides, the pathways of GA and age contained 7 and 2 DEGs in Kashan and 3 and 1 DEGs in accession Viroflay. More importantly, the pathways of vernalization contained 8 DEGs only in the accession Viroflay.

### Transcription factors implicated in spinach bolting

Due to the fact that transcription factor families are associated with the floral development process, we performed a detailed analysis of differential transcription factors to provide further insights into the complex molecular mechanism underlying the bolting.

**Fig. 4:**
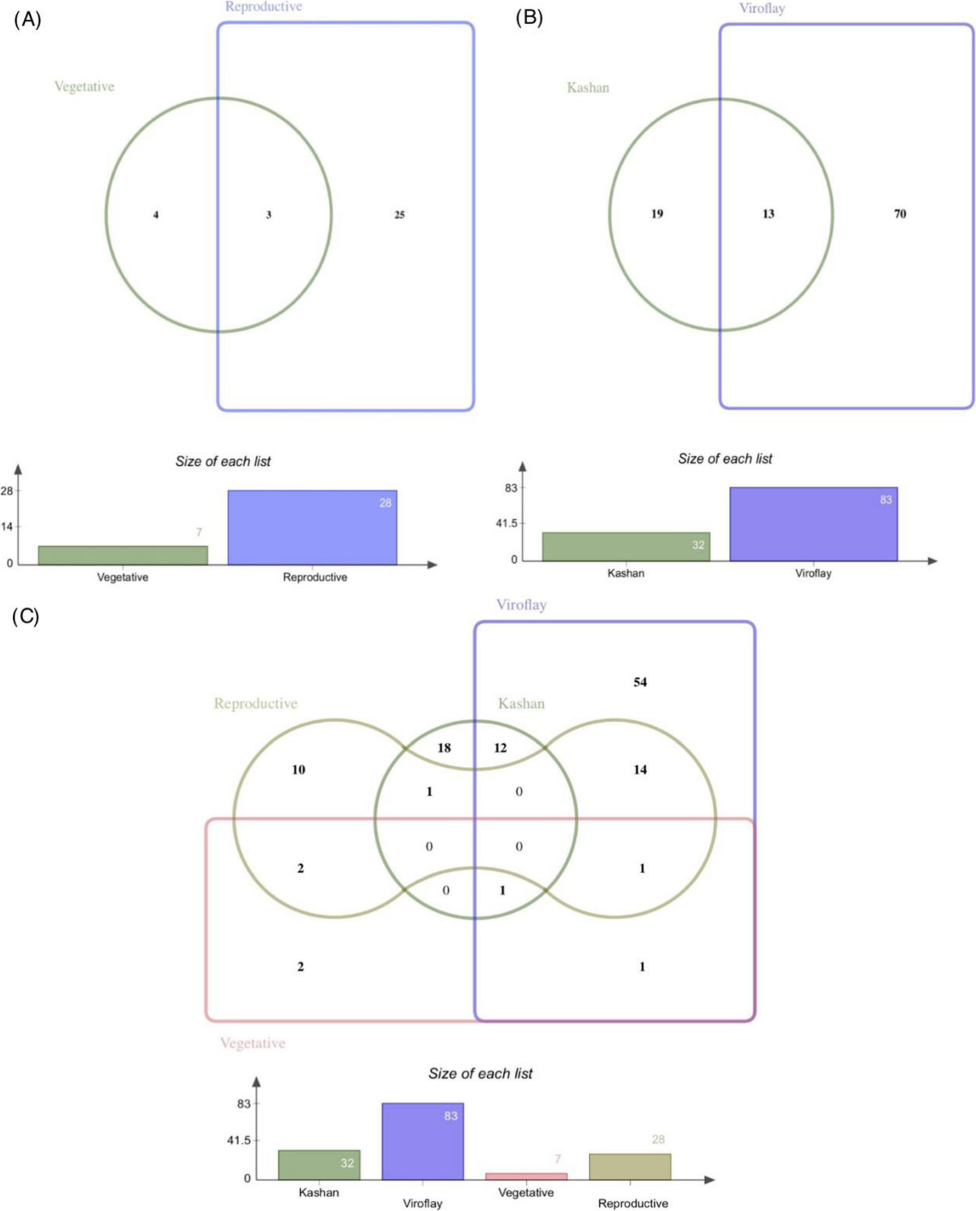
Venn diagram showing the distribution of unique and common differentially expressed TFs among comparisons. The number of TFs commonly shared between stages in the accessions comparison (**a**), between accessions under stages comparison (**b**) and both stages and accessions comparison (**c**)

**Fig. 5:**
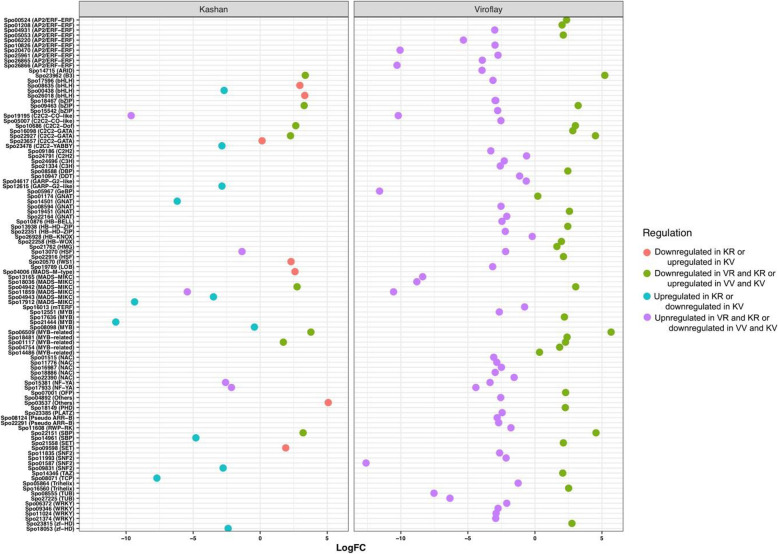
A scatter plot of differentially expressed TFs predicted from the stages comparison in each accession. The y-axis represents the name of TFs, and the x-axis represents the values of the log-fold change. KV, KR, VV, and VR represent Kashan-vegetative, Kashan-reproductive, Viroflay-vegetative, and Viroflay-reproductive, respectively

**Fig. 6:**
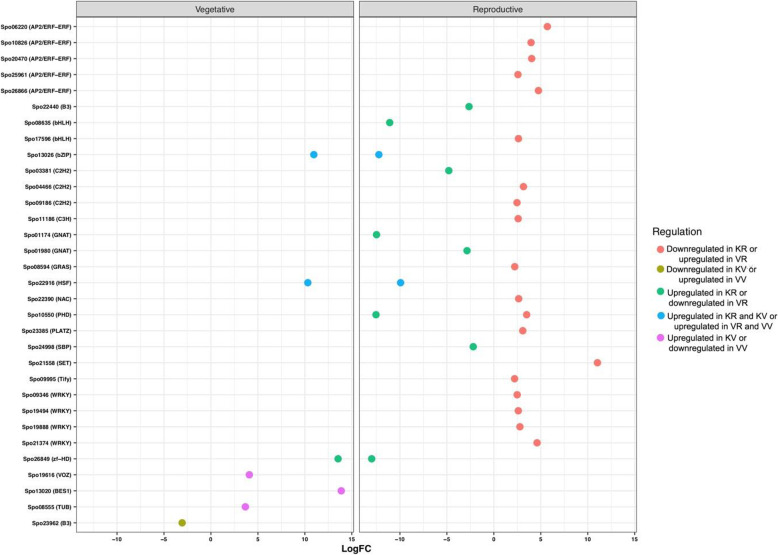
A scatter plot of differentially expressed TFs predicted from accessions comparison in each stage. The y-axis represents the name of TFs, and the x-axis represents the values of the log-fold change. KV, KR, VV, and VR represent Kashan-vegetative, Kashan-reproductive, Viroflay-vegetative, and Viroflay-reproductive, respectively

Among all DEGs identified in the comparison of vegetative and reproductive stages of each accession, a total of 32 and 83 differentially expressed TFs were found in accessions Kashan and Viroflay, respectively. Of these, 13 TFs were commonly expressed in both accessions (Fig. 4a). In the accession Kashan, C2C2-GATA and MADS were the TF families with the most members (5 genes; Fig. 5), MYB was the second-largest TF family with 4 gene members, followed by the bHLH (3 genes). The genes encoding AP2/ERF (10 genes) accounted for the largest proportion of TFs in the accession Viroflay, followed by genes encoding MYB (7 genes), C2C2, and NAC (5 genes). In both accessions, most TFs were upregulated in the reproductive stage compared with the vegetative stage. With respect to the accessions comparison in each stage, 7 and 28 TFs were differentially expressed in vegetative and reproductive stages, respectively (Fig. 4b). The TF families represented by the largest numbers of differentially expressed members were the AP2/ERF (5 genes), WRKY (4 genes), and C2H2 (3 genes) in the reproductive stage (Fig. 6). The majority of TFs were significantly upregulated in the accession Viroflay. The distribution of unique and common TFs identified in each pairwise comparisons of stages and accessions was shown in Fig. 4c.

### Gene set enrichment of predicted-DEGs

**Fig. 7:**
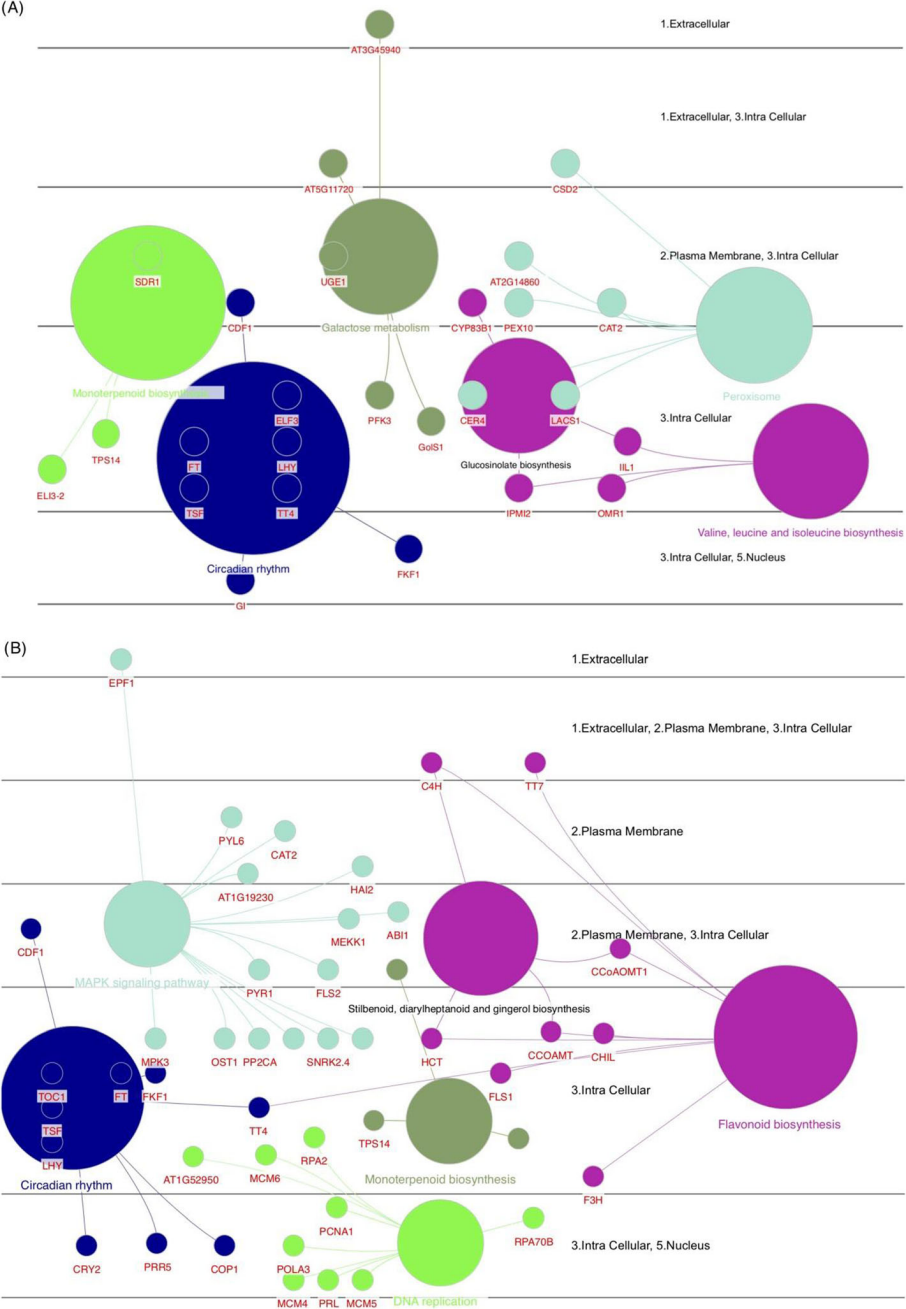
The gene set enrichment analysis of DEGs in Kashan (**a**) and Viroflay (**b**). The enrichment shows only significant pathways (the biological processes, *p*-value ≤0.05). The values of *p* ≤ 0.05 indicate the node size. The color represents various molecular pathways involved in the enrichment analysis of the identified DEGs

The gene set enrichment analysis of the DEGs showed that the circadian rhythm and monoterpenoid biosynthesis were common significantly enriched terms in both accessions (Fig. 7). In circadian rhythm, several core genes such as CYCLING DOF FACTOR 1 (CDF1), FT, TWIN SISTER OF FT (TSF), LATE ELONGATED HYPOCOTYL (LHY), F-Box 1 (FKF1), and TRANSPARENT TESTA 4 (TT4), were identified in both accessions. This term also included two flowering-related genes including GI and ELF3 in the accession Kashan (Fig. 7a), while CONSTITUTIVE PHOTOMORPHOGENIC 1 (COP1), PENTATRICOPEPTIDE REPEAT-CONTAINING PROTEIN 5 (PPR5), and CRYPTOCHROME CIRCADIAN REGULATOR 2 (CRY2) genes were unique in the accession Viroflay (Fig. 7b). Based on the functional analysis results, we detected a few terms such as the MAPK signaling pathway and the flavonoid biosynthesis unique to the accession Viroflay and DNA replication and galactose metabolism unique to the accession Kashan, which suggests the transition from vegetative to reproductive stages may be affected by genes involved in these pathways.

### Validation of differential gene expression using qRT-PCR

**Fig. 8:**
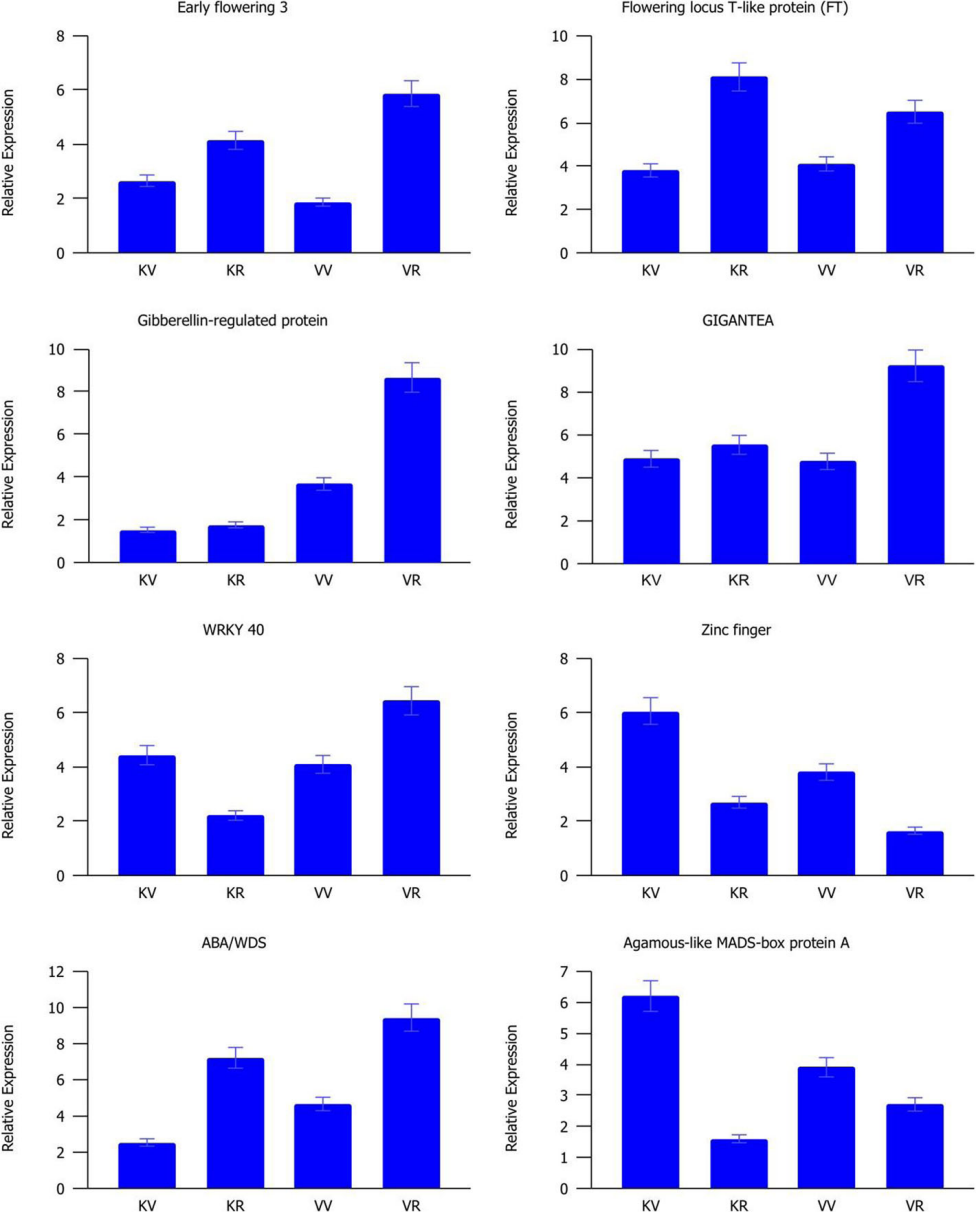
The relative expression of selected genes determined by qPCR in two accessions Kashan and Viroflay at two vegetative and reproductive development stages. KV, KR, VV, and VR represent Kashan-vegetative, Kashan-reproductive, Viroflay-vegetative, and Viroflay-reproductive, respectively. Here the data represented are relative quantification (RQ) values of gene expression

To validate the results of differential expression analysisresults, the relative expression of eight flowering-related genes was examined by qRT-PCR (Fig. 8). Overall, all the selected genes exhibited the same patterns that were consistent with the RNA-seq data, validating positive correlations between the qPCR and RNA-Seq results (Fig. S5). GI, FT, ELF3, and ABA/WDS genes were significantly upregulated in the reproductive stage of both accessions against the vegetative stage, while Agamous-like protein and Zinc finger were downregulated in the reproductive stage. Expression of WRKY 40 was downregulated in the reproductive stage of accession Kashan whereas indicated an opposite expression pattern (as upregulated in reproductive stage) in accession Viroflay. Gibberellin-regulated protein significantly upregulated during the transition from the vegetative to reproductive stage only in accession Viroflay.

### SNP calling

In order to identify SNPs associated with the bolting and reveal the effects of SNPs on the functionality of the corresponding genes, we used RNA-seq data of two spinach accessions to identify SNPs and variable genomic regions. In terms of numbers, a total of 168,849 SNPs were generated from 12 libraries using the GATK pipeline. Of these, 59,022 SNPs remained after filtering using cut-off values. Further, these high-quality SNPs were screened for both kinds of homozygous and heterozygous variants, which remained the 3397 SNPs (Table 1). After exploring, a large number of homozygous reference SNPs were identified from Viroflay (1829 SNPs), which was significantly higher than Kashan (550 SNPs), indicating Viroflay is more closely related to genome reference than the accession Kashan. In contrast, the large rate of heterozygous SNPs in Kashan (2727 SNPs) indicates a higher genetic variability in this accession compared to Viroflay.

**Table 1:** High-quality SNPs screened for both kinds of homozygous and heterozygous variants

Variant type	Kashan reproductive	Kashan vegetative	Viroflay reproductive	Viroflay vegetative
Homozygous reference (0/0)	550	550	1829	1829
Heterozygous (0/1)	2727	2727	670	670
Homozygous alternative (1/1)	130	130	898	898

**Fig. 9:**
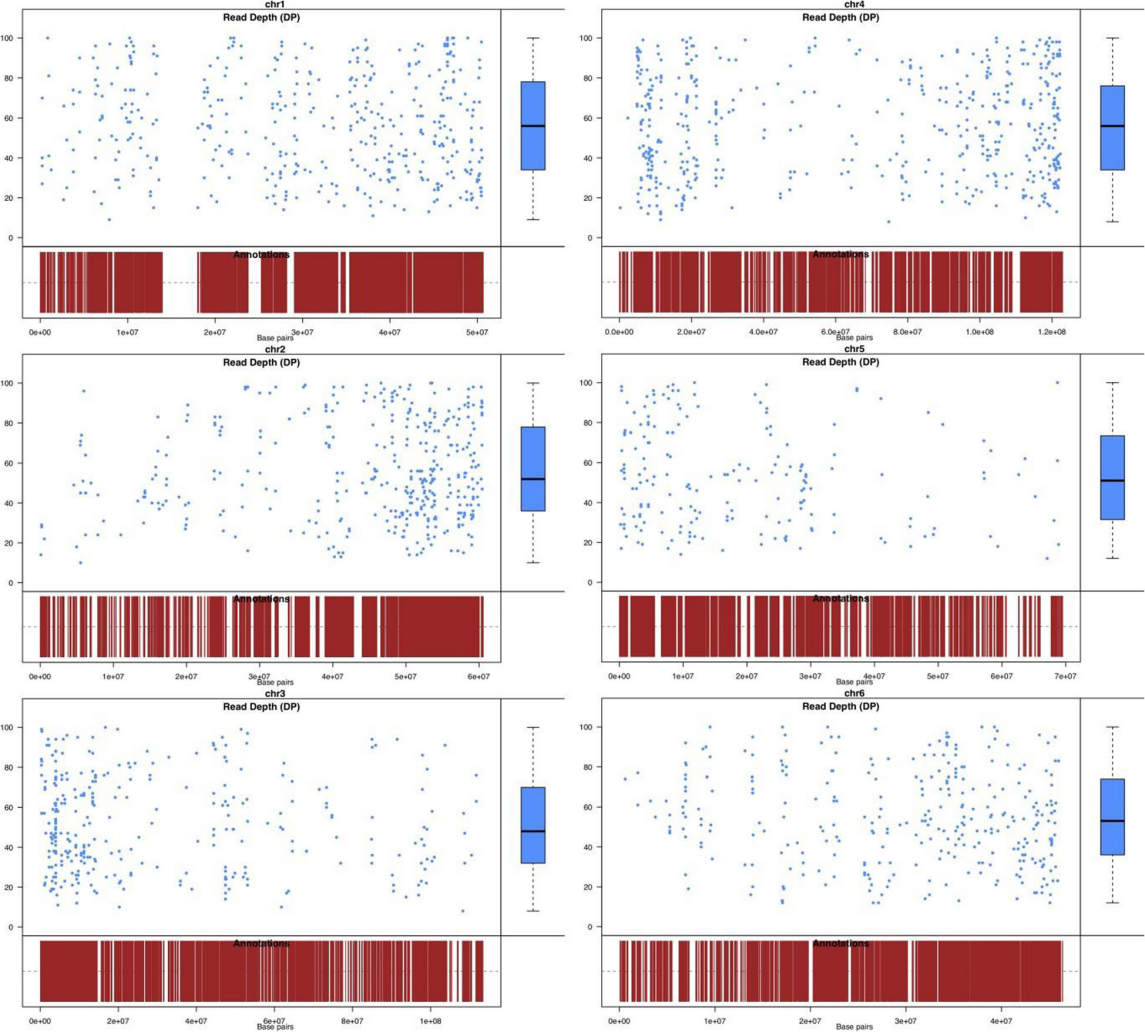
Chromosome-wise distribution of high-quality SNPs in spinach

In further investigation, the SNPs were functionally annotated for obtaining a comprehensive view of the genes associated with the SNPs, giving a total of 3362 annotated SNPs using SnpEff. Of these, 2152 SNPs were distributed across all six chromosomes (Fig. 9). According to the impact results of the SNPs on the functionality of the genes, the vast majority of the variants were categorized into modifier impact (79.12%), followed by low (11.08%), moderate (9.36%), and high (0.42%). It is remarkable the large proportion of variants with modifier effect were observed as downstream (25.84%), upstream (17.1%), intergenic (14.61%), and intron (10.3%) gene variants (Fig. S6), which indicates the presence of these variants in unannotated exons and/or non-coding regions. The low effect SNPs mostly occurred as UTR (11.77%), and synonymous (8.3%) variants, whereas the moderate impact SNPs were observed as missense (11.03%) variants. Moreover, the high impact SNPs were mostly identified as splice variants (0.46%).

Additionally, the SNPs have also been classified according to GO terms for obtaining a comprehensive view of the genes associated with the SNPs. The functional annotation of the filtered SNPs revealed the association of SNPs with genes involved in numerous vital biological processes. Among the biological process annotated, a significant number of SNPs were linked to genes involved in the flowering process, including photosynthesis (49 genes), carbohydrate metabolic process (200 genes), reproduction (77 genes), flower development (55 genes), pollination (11 genes), embryo development (30 genes), and post-embryonic development (70 genes).

## Discussion

The timing of bolting is essential for productivity success in vegetative crops such as spinach. Our past investigation to screen accessions affirmed that bolting time in Kashan (accession with early-bolting phenotype) varies from that of Viroflay with a high potential in the production (accession with late-bolting phenotype) [30]. By studying plants’ genome and transcriptome, researchers have provided much essential information on key genes and mechanisms associated with bolting and flowering regulation. Hence, to determine molecular networks and bolting-related genes in spinach, we investigated the most prominent gene expression changes in leaf tissues of two accessions at different developmental stages using RNA-seq technology. Therefore, a significant number of bolting and flowering related genes which differentially expressed between stages and accessions were recongized by transcriptome analysis., to gain deeper insight into the molecular events that regulate bolting in spinach, DEGs were utilized for GO, KEGG, TFs analyses.

GO terms associated with biological processes for DEGs included ‘metabolic processes’, ‘cellular processes’, and ‘biosynthetic processes’ as the most highly represented terms. Among enrichment terms determined in all comparisons, ‘reproduction’, ‘carbohydrate metabolic process’, ‘generation of precursor metabolites and energy’, and ‘flower development’ were the most related terms, which have roles in the vegetative to reproductive transition. Previous evidence demonstrates the role of carbohydrates in the vegetative to reproductive transition as energy reserves for inflorescence development and signaling molecules [15]. Interestingly, carbohydrates can promote bolting through the up-regulation of flowering promoters [31]. The stringent analyses of genes expression pattern encoding enzymes involved in carbohydrate metabolism indicated a clear differential pattern between stages and accessions. Our integrative results showed that a significant number of carbohydrate-related DEGs (50 genes) were up-regulated in the vegetative stage of Viroflay compared to accession Kashan (16 genes). Among these genes, Fructose-1, 6-bisphosphate aldolase (FBA) that is involved in glycolysis, gluconeogenesis, and the Calvin cycle with significant roles in regulating growth and development processes [32, 33] was found to be up-regulated in accession Viroflay at the vegetative stage. It has been concluded that increase of activity of FBA enhances photosynthetic capacity, growth, and plant productivity or biomass [34–36], which is consistent with the high-yield of accession Viroflay as reported in the previous studies [30] and a higher number of DEGs associated with “photosynthesis” term in Viroflay under present study. Among genes involved in carbohydrate metabolism, TREHALOSE-6-PHOSPHATE SYNTHASE 1 (TPS1), which catalyzes the formation of trehalose-6-phosphate (T6P), has been proposed to function as a proxy for carbohydrate status and a signal that coordinates the induction of flowering in plants [37–39]. In this way, Alpha, alpha-trehalose-phosphate synthase (TPS) gene with having important roles in sucrose and starch synthesis [37, 38], and TPS1 were highly up-regulated in both vegetative and reproductive stages of accession Kashan, while in Viroflay showed an increase in expression during the transition from vegetative to reproductive growth. Similarly, there was no obvious difference in sucrose synthase activity during growth development in Kashan (a high expression in both stages), while its expression level increased during the transition from vegetative to reproductive in accession Viroflay. These results demonstrate the potential role of these genes in the flowering delay of accession Viroflay and their target for genome manipulation and genetic improvement in spinach to obtain late-bolting varieties. The GO annotation results of DEGs retrieved FLOWERING PROMOTING FACTOR 1 (FPF1), Transcription factor MADS-box, ELF, and GI as the most important genes in flower development subcategory, which highly expressed in accession Kashan. Previous studies have indicated that FPF1 gene is involved in the promotion of flowering [40], thereby initiating bolting. We found that FPF1 was highly expressed in the vegetative stage of Kashan while it didn’t show any expression change during growth in accession Viroflay, indicating that probably FPF1 has a stronger effect on the early bolting of accession Kashan. On the other hand, the transcription factor MADS-box showed a sharp increase in expression during the transition from vegetative to the reproductive stage in Kashan, whereas it was considered a non-significant gene in accession Viroflay. In previous reports, MADS-box transcription factors have been characterized in various species such as *A. tequilana* [15], radish [16], carrot [17], and lettuce [18] as a major group of regulators controlling floral transition and therefore exhibited differential expression patterns at different developmental stages and in different tissues. GI is a unique plant protein involved in multiple biological functions, including photoperiodic flowering, control over the circadian rhythm, and light and hormone signaling [41–43]. Overexpression of GI in wheat [44] and barley [45] modified flowering time, leading to early flowering phenotype. According to genetic data, COP1 and ELF3 control flowering by regulating GI stability, so that ELF3 allows COP1 to interact with GI, resulting in GI degradation [43, 46]. In our study, ELF3 and GI were up-regulated in the reproductive stage while COP1 was up-regulated in the vegetative stage of both accessions. Although all three genes showed a similar expression trend during the transition from the vegetative stage to the reproductive stage in both accessions, the accessions comparison in each stage exhibited their different expression level. The results indicated a lower expression level for ELF3 and GI genes in the vegetative stage of accession Viroflay against Kashan. In contrast, COP1 showed a higher expression in a similar stage and accession, suggesting low expression of COP1 in spinach causes early flowering, possibly by increased accumulation of transcripts of floral inducers like GI. Additionally, FLAVIN-BINDING, KELCH REPEAT, F BOX protein 1 (FKF1), a clock-controlled gene was found to be up-regulated in both accession reproductive stages [47]. FKF1 has been reported to promote flowering by making a complex with GI that up-regulates CO and FT [41, 48]. Indeed, expression levels of these key integrator genes precisely adjust the expression of floral specific genes and ultimately determine the exact FT so that the low expression of FKF1 in the vegetative stage of accession Viroflay may cause late flowering in this accession.

Besides the core flowering-related genes, TFs have been reported to be an essential regulators group to control bolting and flowering by affecting the expression of flowering pathways genes and integrating endogenous/exogenous signals [49–51]. Since TFs families could also provide insights into comparative transcriptional status between genotypes or cultivars, we performed a detailed analysis of differentially expressed transcription factors. In previous investigations, many specifically expressed TFs such as the C2C2-GATA, AP2/ERF, MADS-box, MYB, bHLH, and NAC have distinguished during the flowering process [15, 16, 18, 51–53]. Researchers have demonstrated that GATAs have an essential role in regulating the flowering time so that two GATA factors GNC and CGA1 in *A. thaliana* directly repress SOC1 expression and thereby repress flowering [53]. In our study, the GATA TFs were overrepresented as up-regulated genes during the vegetative phase of the accession Viroflay but were not identified as DEGs. AP2/ERF superfamily members have a potential impact to positively/negatively regulate various processes such as control of metabolism, growth, and development, as well as flowering regulation through photoperiod pathway [54]. However, information about how these AP2/ERFs regulate flowering time is limited. Thus, only a few examples of AP2/ERFs in other species have been discussed concerning flowering or bolting. For example, the over-expression of seven AP2 genes in *Glycine max* leads to early flowering [55], or the expression of miR172, which targets the transcripts of AP2 TFs, inhibits flowering [56]. The transcriptomic profiles obtained in this study indicated seven AP2/ERF TFs were downregulated in vegetative tissue of accession Viroflay, whereas 17 AP2/ERF TFs were upregulated in the same tissue of accession Kashan, but their mRNA levels did not show significant changes against the reproductive stage. From the results presented in this study and previous investigation, we concluded that the different expressions of AP2 TFs between stages and accessions should be responsible for controlling the flowering time. Among TF families, MADS-box (MIKC-type) is introduced to be the most important flowering-related TFs in plants with higher expression in bolting sensitive lines, cultivars, or species [15, 56–58]. Interestingly, AGAMOUS has known a subfamily of MADS-box genes that regulate the transcription of two important flowering-time regulators, FLC, and FT [59]. In our study, AGAMOUS-like MADS-box protein recorded higher expressions in the vegetative stage of accession Kashan, supporting the bolting sensitive phenotype in this accession. According to overall expression patterns of TFs in this study and previous researches, we concluded GATA, MADS-box, and MYB TFs in spinach produce similar transcriptomic profiling with those vegetative species that bolting and flowering greatly limit production.

Finally, at the latest consideration, we aimed to investigate the SNPs related to bolting in early and late flowering accessions of spinach using transcriptome data. The revelation of SNPs from the transcriptome data yielded a sum of 168,849 SNPs, which upon intense filtering diminished to 59,022 polymorphic SNPs over the accessions. Furthermore, these high-quality SNPs were screened for both kinds of homozygous and heterozygous variants, which resulted in higher homozygous SNPs in Viroflay against Kashan. Subsequently, from the filtered 3397 SNPs, 2152 were distributed across the length of the six chromosomes, which are useful for the generation of marker-assisted backcross marker probes. However, the number of SNPs was varied among the chromosomes; with the higher number in chromosome 4 followed by chromosome 1 and the lowest in chromosome 5. Additionally, in the current investigation, the SNPs have practically been annotated for obtaining a comprehensive view of the genes associated with the SNPs. The functional annotation results indicated the association of SNPs with genes involved in numerous biological processes. Among the biological processes annotated, the majority of SNPs were linked to genes involved in the cellular process, biosynthetic process, and metabolic process, similar to the functional annotations of SNPs reported in radish, onions, and capsicum [20, 52, 60, 61]. Interestingly, a significant number of SNPs were also linked to genes involved in the flowering process, including photosynthesis, carbohydrate metabolic process, reproduction, and flower development. More investigation indicated that the identified SNPs were mostly associated with the four major transcription factors such as Zinc finger, AP2/ERF, bHLH, and WRKY. In detail, SNPs in the genes involved in the flowering pathways were identified. Among these genes, INDUCER OF CBF EXPRESSION 1 (ICE1), HISTONE-LYSINE N-METHYLTRANSFERASE, FKF1, LUX ARRHYTHMO (LUX), PHYTOCLOCK 1 (PCL1) associated with the vernalization pathway and circadian clock were identified as key genes involved in the flowering-time control.

## Conclusion

In the present study, we investigated transcription changes of two spinach accessions in the transition from vegetative growth to the reproductive stage by using RNA-seq. We identified a set of DEGs associated with the vernalization, photoperiod/circadian clock, gibberellin, autonomous, and aging pathways. These results demonstrated the potential role of some of these specific genes in the flowering delay of accession Viroflay and their sufficient target for genome manipulation and genetic improvement in spinach to obtain late-bolting varieties.

## Methods

### Plant material

According to our previous study [30], two accessions including Kashan and Viroflay were selected and used as early and late-bolting spinach samples, respectively. In the previous study [30], the vegetative characteristics of 44 spinach accessions were evaluated based on descriptors investigated by Bioversity International Plant Genetic Resources Institute. According to the results of this research, two accessions Viroflay and Kashan were placed in the group of late and early flowering spinach, respectively. Indeed, maximum variation for the trait of “days to flowering” was found between accessions Viroflay (87 days) and Kashan (43 days). To make a stable condition and eliminate influential environmental factors, seeds of each accession were sown in plastic pots (15 cm diameter, 25 cm high) with sterilized soil and grown in a growth chamber under spring growth conditions for 3 months at Isfahan University of Technology, Isfahan, Iran, in March 2018. In this condition, growth period was also calculated as days to flowering, resulted in 83 and 43 days for accessions Viroflay and Kashan, respectively. To obtain these samples the permissions were not necessary. The formal identification of the plant material was undertaken by the herbarium of Agricultural and Natural Resources College, University of Tehran, and no voucher specimens were collected and deposited in the collection (it is not necessary as we don’t describe a novel species). We also stated that the field studies were in compliance with local legislation of Iran in the experimental greenhouse and growth chamber of Isfahan University of Technology, Isfahan, and no specific licences were required.

### RNA isolation, library construction, and sequencing

Total RNAs were extracted from leaf samples of two accessions Kashan and Viroflay at vegetative (four-leaf stage) and reproductive (when 50% of the plants produced flower-stalks) stages in three biological replicates using DENAzist column RNA isolation kit. Indeed, stages were selected so that the majority of differentiation takes place between stages (Days to flowering). Each sample was a pool gathered from at least ten plants in order to decrease the variance caused by interindividual differences in gene expression. The RNAs were quantified on an agarose gel, and the quality was determined based on absorbance ratios (260/280 nm and 260/230 nm) using the NanoDrop spectrophotometer (NanoDrop Technologies). Furthermore, subsequent quality control by using a QC Bioanalyzer, cDNA library preparation, and sequencing was performed at the Personalbio (Shanghai, China), according to the manufacturer’s recommendation. The sequencing was done on an Illumina platform with 150 bp paired-end readers. The reads obtained from sequencing were deposited in the NCBI Sequence Read Archive (https://www.ncbi.nlm.nih.gov/) under accession number PRJNA630139.

### Read mapping, expression level calculation, and mining of DEGs

Clean paired-end reads from each of the samples were individually mapped versus the spinach genome assembly version 1 [28] using STAR v2.7.1 [62] software, and then transcripts assembled by the StringTie v2.0.6 [63] with default parameters (without the ‘-e’ option). The assembled transcripts were merged using StringTie’s merge function to create a unique set of transcripts for stages and accessions. The mapped reads were assembled again using Stringtie software with the merged transcripts as a direction, and the ‘-e’ and ‘-B’ options were used to restrict novel transcript prediction and generate an input file for DEGs identification. For differential expression analysis, gene read-count data matrices were produced with python script prepDE.py for stages and accessions. Finally, DEGs were identified through the IDEAMEX website [64], using the EdgeR [65], DESeq2 [66], and NOISeq [67]. The threshold to judge the significance of gene expression differences was “FDR ≤ 0.05, the absolute value of logFC> = 2 and CPM = 1”.

### Function investigation of DEGs

Genes classification and transcription factors identification analysis were conducted on all DEGs using gene functional classification tools and the latest genomic reference information of *S. oleracea* in SpinachBase (http://spinachbase.org) [29]. For gene set enrichment analysis, all DEGs were mapped to the protein sequences source of *Arabidopsis* (Araport11_genes.201606.pep.fasta) using the BLAST search. This is because of the well-maintained and annotated *Arabidopsis* genome. Finally, the ClueGO [68] plug-in v3.7.2 of Cytoscape software [69] was used to identify significant pathways and to visualize genes in functionally grouped networks.

### Quantitative real-time polymerase chain reaction

To validate the expression pattern of bolting and flowering-related genes, quantitative real-time polymerase chain reaction (qRT-PCR) was applied to quantitatively measure the eight candidate genes expression in leaf tissues of two accessions at different developmental stages. In this way, gene-specific primers (Table 2) with melting temperature (Tm) 60 °C were designed using the Primer3 software (http://frodo.wi.mit.edu/primer3/). qRT-PCR reactions were performed in three technical replicates using an ABI system (ABI ViiA 7 Real-time PCR) in a 20 μL final volume, containing 10 μL SYBR Green Master Mix (BioFACT, Korea), 2 μL of diluted cDNA, and 1 μL of each primer (10 μM) in conjunction with adding PCR-grade water. The qPCR experiment was carried out based on a thermal program of 5 min at 95 °C, 40 cycles of 10 s at 95 °C, 20 s at the specific annealing temperature for each primer, 20 s at 72 °C, and finally a melting curve program. The statistical analysis of gene expression was carried out using the 2 − ∆∆Ct method [70] by using *Actin* and *GAPDH* as internal reference (housekeeping) genes.

**Table 2:** Genes and primers set used for qRT-PCR analysis

Gene IDs	Gene Names	Primers Sequence	Product Size
Spo04360_F	GIGANTEA	CATTTCCATTCGAGTCTTCTCC	197
Spo04360_R		CATCAGCCCTTGAACTTTTACC	
Spo14415_F	Early flowering 3	TCCTCTGTCAACACAATTCCAC	203
Spo14415_R		TAAGCAGGATTCATGACAGGTG	
Spo10686_F	Zinc finger	GCCAGTTCCATGTTTTCCTTAC	198
Spo10686_R		GAATGTTTTCCAAGGGTGGTAG	
Spo11981_F	Flowering locus T-like protein (FT)	GAGTACTTGCATTGGTTGGTGA	214
Spo11981_R		AGCCGAGGTTGTATATTTCAGC	
Spo07804_F	Gibberellin-regulated protein	AATGCTGATCTCTCCGTTATGC	167
Spo07804_R		TCCCTATAACAAGGGCACTCAT	
Spo20053_F	ABA/WDS	ACACAAACAACACTTGGCTGAG	186
Spo20053_R		GCTTCCTTCTTCTGATGATGCT	
Spo19888_F	WRKY 40	AAGCTAAGATCACAAGGGTTGC	220
Spo19888_R		CCTTCATACGTTGCCACTAACA	
Spo04006_F	Agamous-like MADS-box protein A	CGAGTTGACAACCTTGTGTGAT	179
Spo04006_R		TTTGCCTTAGGAACTCCTCTTG	
Spo21495_F	Glyceraldehyde 3-phosphate dehydrogenase (GAPDH)	CGTGTCAGTTGATTTCAGGTGT	223
Spo21495_R		GTTGTCCTTGCAGAAATCTTCC	
Spo05970_F	Actine 11	AGTCCCCATTTACGAAGGGTAT	221
Spo05970_R		CGGAAGAGCTAGTTTTTGCAGT	

### SNPs detection from RNA-seq data

SNPs were called from transcriptome data by a Joint genotyping method [71] using the Genome Analysis Toolkit (GATK 4.1) in accordance with the Best Practices workflow for variant calling on RNA-seq data. We first used the two-pass mapping strategy (−twopassMode Basic) with STAR aligner [62] to generate coordinate-sorted BAM files and then read groups were added to BAM files with the Picard tool AddOrReplaceReadGroups (https://broadinstitute.github.io/picard/). Next, duplicated reads were marked with Picard tools, so that GATK tools could automatically ignore them. Subsequently, SAMtools v1.10 [72] was applied to merge BAM files belong to each stage and the recommended Split’N’Trim and indel realignments steps were also performed on each merged BAM file. In the next step of this approach, the potential variants were called using HaplotypeCaller algorithm with the –ERC GVCF mode, leading to the production of gVCF files. Then, variants were called through a Joint Genotyping analysis from all gVCF files. As recommended by the GATK Best Practices, variant filtering was performed at this step with the following options: --filter-expression “QD < 2.0” --filter-name “SNPQDFilter” --filter-expression “FS > 30.0” --filter-name “SNPFSFilter” --filter-expression “MQ < 40.0” --filter-name “SNPMQFilter” --filter-expression “MQRankSum < -12.5” --filter-name “SNPMQRSQFilter” --filter-expression “ReadPosRankSum < -8.0” --filter-name “SNPRPRSFilter” --filter-expression “HaplotypeScore > 13.0” --filter-name “SNPHSFilter” --filter-expression “DP>100 || DP<5”. Next, this VCF file was fed into VCFtools v0.1.16 [73] to remove indels, and finally, SnpSift v4.3 [74] was used for pulling out specific genotype combinations.

## Supplementary Information

**Additional file 1: Table S1** Summary of transcriptome sequencing results from 12 spinach samples. **Table S2** Summary of STAR alignment rates from 12 spinach samples. KV, KR, VV, and VR represent Kashan-vegetative, Kashan-reproductive, Viroflay-vegetative, and Viroflay-reproductive, respectively. **Fig. S1.** Distribution of DEGs. (A) The discovered DEGs in the vegetative stages comparison (B) The discovered DEGs in the reproductive stages comparison. The X-axis represents the location of DEGs on chromosomes. Y-axis is the log2 fold change for each. **Fig. S2.** Distribution of DEGs across six chromosomes. (A) The discovered DEGs from the stages comparison of accession Kashan (B) The discovered DEGs from the stages comparison of accession Viroflay. The X-axis represents the location of DEGs on chromosomes. Y-axis is the log2 fold change for each. **Fig. S3.** Gene classification of identified DEGs. The histogram shows the classification of DEGs under the molecular function category for all pairwise comparisons. **Fig. S4.** Gene classification of identified DEGs. The histogram shows the classification of DEGs under the cellular component category for all pairwise comparisons. **Fig. S5.** Gene expression correlation between RT-qPCR and RNA-seq data (Log2 values of the fold change). The Pearson correlation coefficients and linear regression line are indicated. **Fig. S6.** The summary statistics of variant effects by the type and region.

**Additional file 2.**

## Data Availability

All RNA-Seq data were deposited in the NCBI SRA database under the project PRJNA630139 (https://www.ncbi.nlm.nih.gov/bioproject/PRJNA630139).

## Abbreviations

DEGs: Differentially expressed genes
qRT-PCR: Quantitative real-time polymerase chain reaction
TFs: Transcription factors
GO: Gene ontology
SOC1: Suppressor of Overexpression of CO1
FT: Flowering locus T
LFY: Leafy
CO: Constans
FLC: Flowering locus c
SPL1: Squamosa promoter binding-like protein 1
TPS1: Trehalose-6-phosphate synthase 1
ICE1: Inducer of cbf expression 1
FPF1: Flowering promoting factor 1
FKF1: Flavin-binding, kelch repeat, F box protein 1
COL1: Constans-like 1
ELF3: Early flowering 3
AGL: Agamous-like
GI: Gigantea
