# Supplementary material for: Transcriptome architecture reveals genetic networks of bolting regulation in spinach
Source: BMC Plant Biol. 2021 Apr 14;21:179. doi: 10.1186/s12870-021-02956-0 (PMC8045288; doi:10.1186/s12870-021-02956-0)
Supplement: Supplementary file 1 — Additional file 1: Table S1 Summary of transcriptome sequencing results from 12 spinach samples. Table S2 Summary of STAR alignment rates from 12 spinach samples. KV, KR, VV, and VR represent Kashan-vegetative, Kashan-reproductive, Viroflay-vegetative, and Viroflay-reproductive, respectively. Fig. S1. Distribution of DEGs. (A) The discovered DEGs in the vegetative stages comparison (B) The discovered DEGs in the reproductive stages comparison. The X-axis represents the location of DEGs on chromosomes. Y-axis is the log2 fold change for each. Fig. S2. Distribution of DEGs across six chromosomes. (A) The discovered DEGs from the stages comparison of accession Kashan (B) The discovered DEGs from the stages comparison of accession Viroflay. The X-axis represents the location of DEGs on chromosomes. Y-axis is the log2 fold change for each. Fig. S3. Gene classification of identified DEGs. The histogram shows the classification of DEGs under the molecular function category for all pairwise comparisons. Fig. S4. Gene classification of identified DEGs. The histogram shows the classification of DEGs under the cellular component category for all pairwise comparisons. Fig. S5. Gene expression correlation between RT-qPCR and RNA-seq data (Log2 values of the fold change). The Pearson correlation coefficients and linear regression line are indicated. Fig. S6. The summary statistics of variant effects by the type and region. [file 12870_2021_2956_MOESM1_ESM.docx]

**Transcriptome architecture reveals genetic networks of bolting regulation in spinach**

Reza Abolghasemi^1^, Maryam Haghighi^1^, Nematollah Etemadi^1^, Shui Wang^2^, Aboozar Soorni^3^*

^1^Department of Horticulture, College of Agriculture, Isfahan University of Technology, Isfahan, Iran

^2^College of Life and Environmental Sciences, Shanghai Normal University, Shanghai, China

^3^Department of Biotechnology, College of Agriculture, Isfahan University of Technology, Isfahan, Iran

*Correspondence: [soorni@iut.ac.ir](mailto:soorni@iut.ac.ir)

**Table S1** Summary of transcriptome sequencing results from 12 spinach samples.

| Sample | Stage | Replicates number | Raw read number | Raw Q30 rate (%) | Trimmed read number | Useful read (%) |
| --- | --- | --- | --- | --- | --- | --- |
| Kashan | Vegetative | 1 | 45884264 | 93.82 | 42744762 | 93.15 |
| Kashan | Vegetative | 2 | 46452936 | 93.85 | 42693790 | 91.9 |
| Kashan | Vegetative | 3 | 44928630 | 94.34 | 41744234 | 92.91 |
| Viroflay | Vegetative | 1 | 50055002 | 93.73 | 46645352 | 93.18 |
| Viroflay | Vegetative | 2 | 44224312 | 94.2 | 41005496 | 92.72 |
| Viroflay | Vegetative | 3 | 49879790 | 94.04 | 46168548 | 92.55 |
| Kashan | Reproductive | 1 | 49837006 | 94.25 | 46267110 | 92.83 |
| Kashan | Reproductive | 2 | 42698438 | 94.14 | 39654632 | 92.87 |
| Kashan | Reproductive | 3 | 46585646 | 93.96 | 43384448 | 93.12 |
| Viroflay | Reproductive | 1 | 47792736 | 94.13 | 44526090 | 93.16 |
| Viroflay | Reproductive | 2 | 44310526 | 94.09 | 41166544 | 92.9 |
| Viroflay | Reproductive | 3 | 46414652 | 93.95 | 43229800 | 93.13 |

**Table S2** Summary of STAR alignment rates from 12 spinach samples. KV, KR, VV, and VR represent Kashan-vegetative, Kashan-reproductive, Viroflay-vegetative, and Viroflay-reproductive, respectively.

| Sample Name | % Aligned | M Aligned |
| --- | --- | --- |
| KR1 | 90.10% | 20.8 |
| KR2 | 91.70% | 18.2 |
| KR3 | 91.50% | 19.9 |
| KV1 | 90.70% | 19.4 |
| KV2 | 89.50% | 19.1 |
| KV3 | 90.40% | 18.9 |
| VR1 | 90.60% | 20.2 |
| VR2 | 91.40% | 18.8 |
| VR3 | 92.20% | 19.9 |
| VV1 | 91.20% | 21.3 |
| VV2 | 91.60% | 18.8 |
| VV3 | 91.00% | 21 |

**
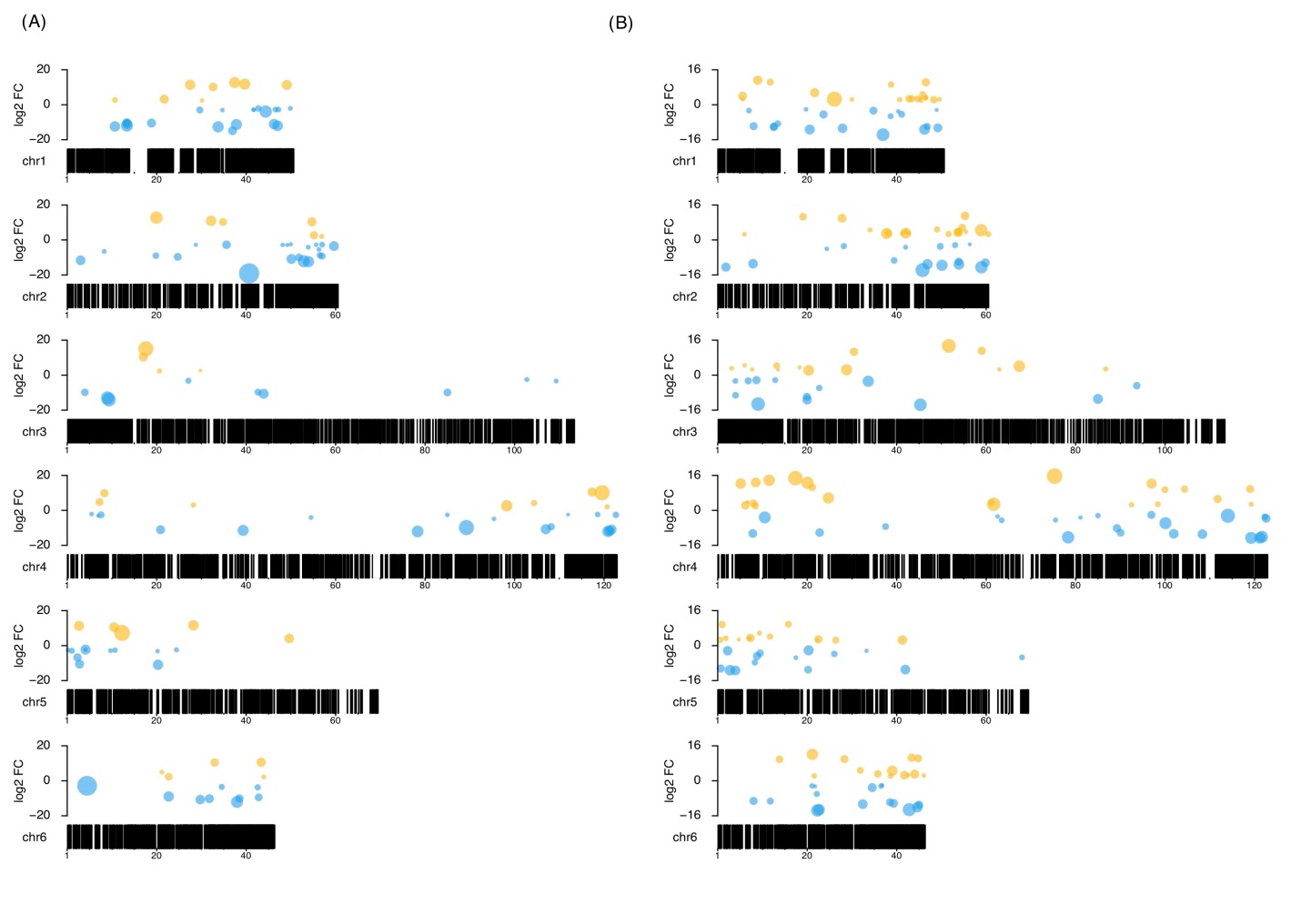
**

**Figure S1: Distribution of DEGs. (A) The discovered DEGs in the vegetative stages comparison (B) The discovered DEGs in the reproductive stages comparison. The X-axis represents the location of DEGs on chromosomes. Y-axis is the log2 fold change for each.**

**
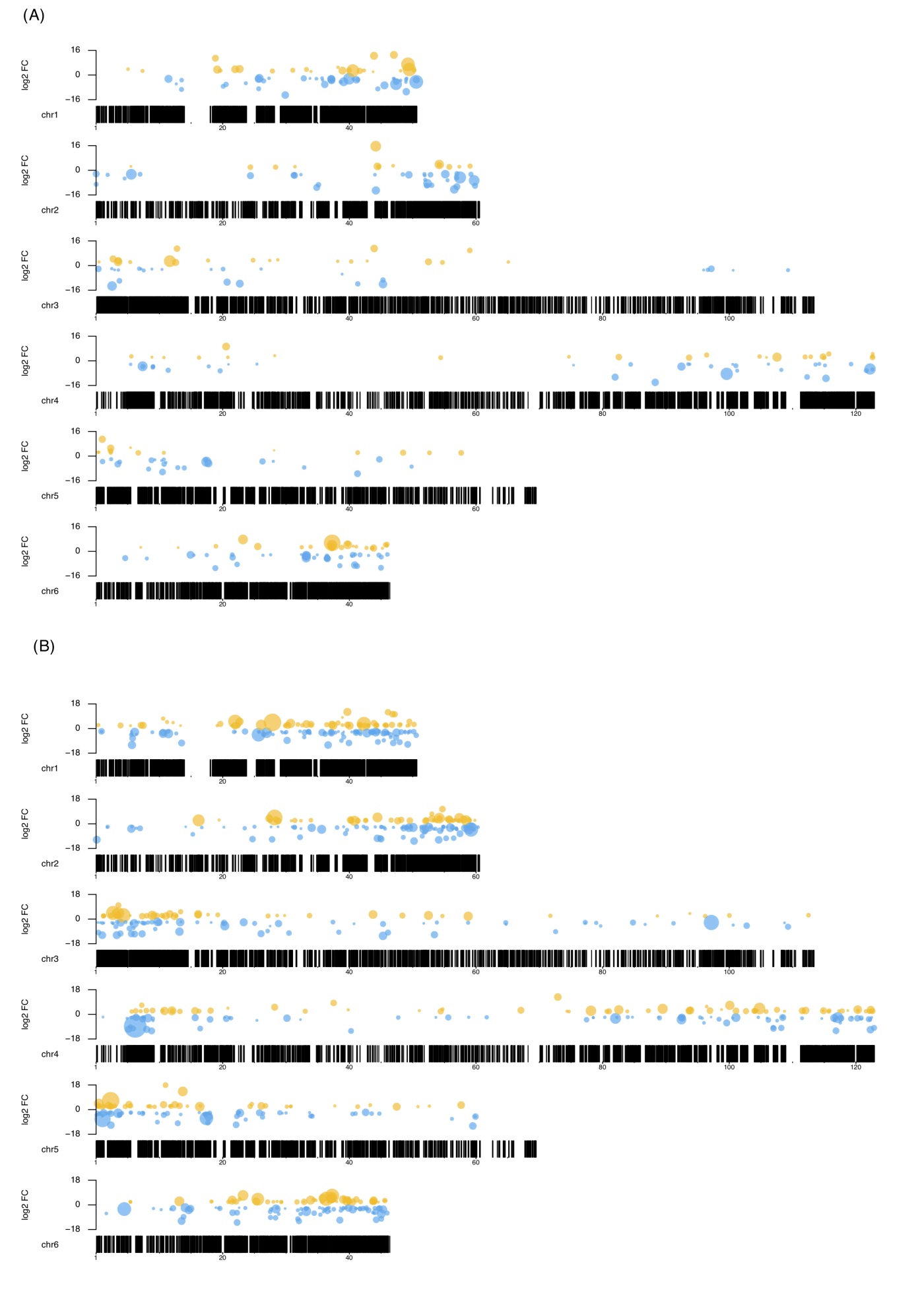
**

**Figure S2: Distribution of DEGs across six chromosomes. (A) The discovered DEGs from the stages comparison of cultivar Kashan (B) The discovered DEGs from the stages comparison of cultivar Viroflay. The X-axis represents the location of DEGs on chromosomes. Y-axis is the log2 fold change for each.**

**
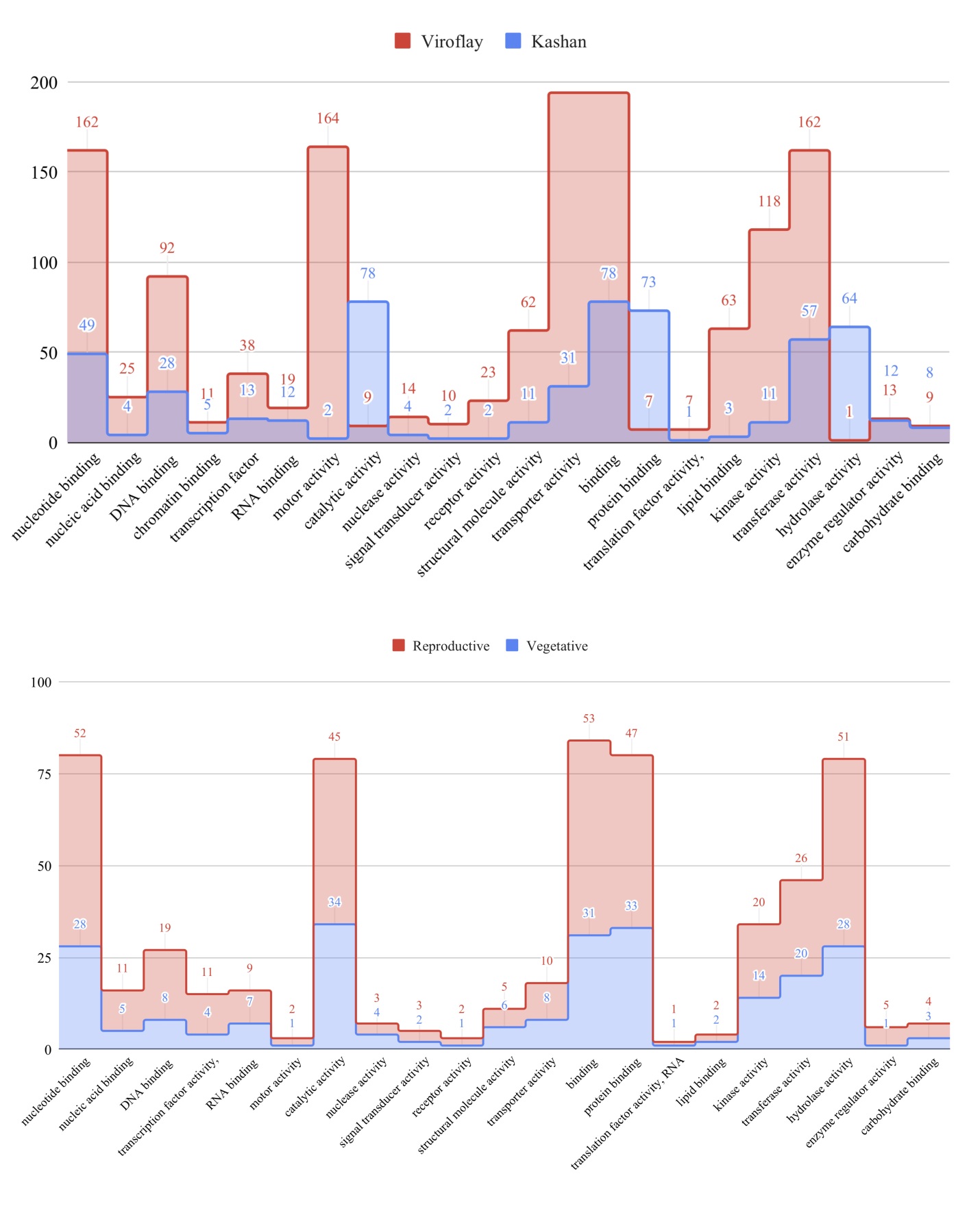
**

**Figure S3. Gene classification of identified DEGs. The histogram shows the classification of DEGs under the molecular function category for all pairwise comparisons.**

**
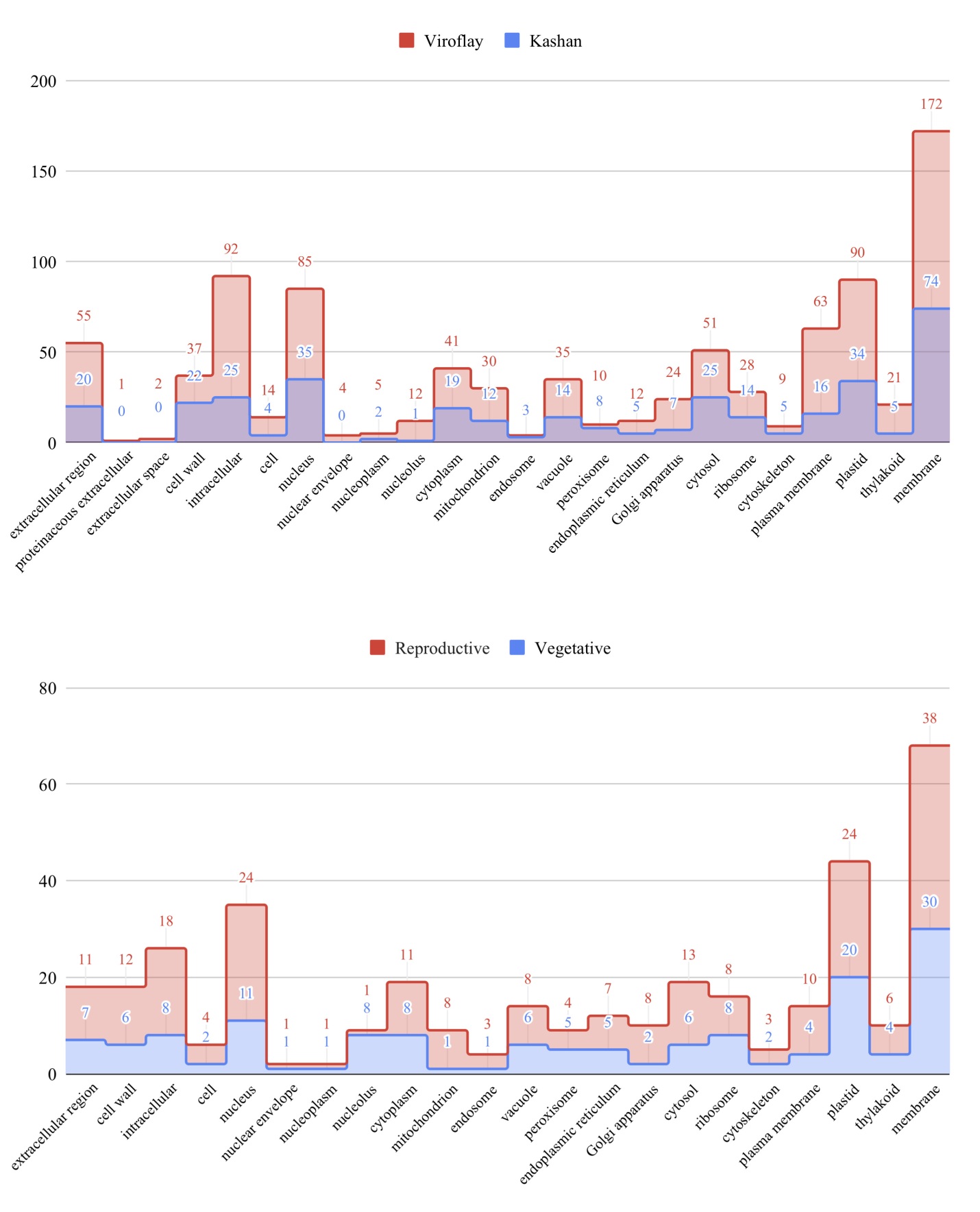
**

**Figure S4. Gene classification of identified DEGs. The histogram shows the classification of DEGs under the cellular component category for all pairwise comparisons**.


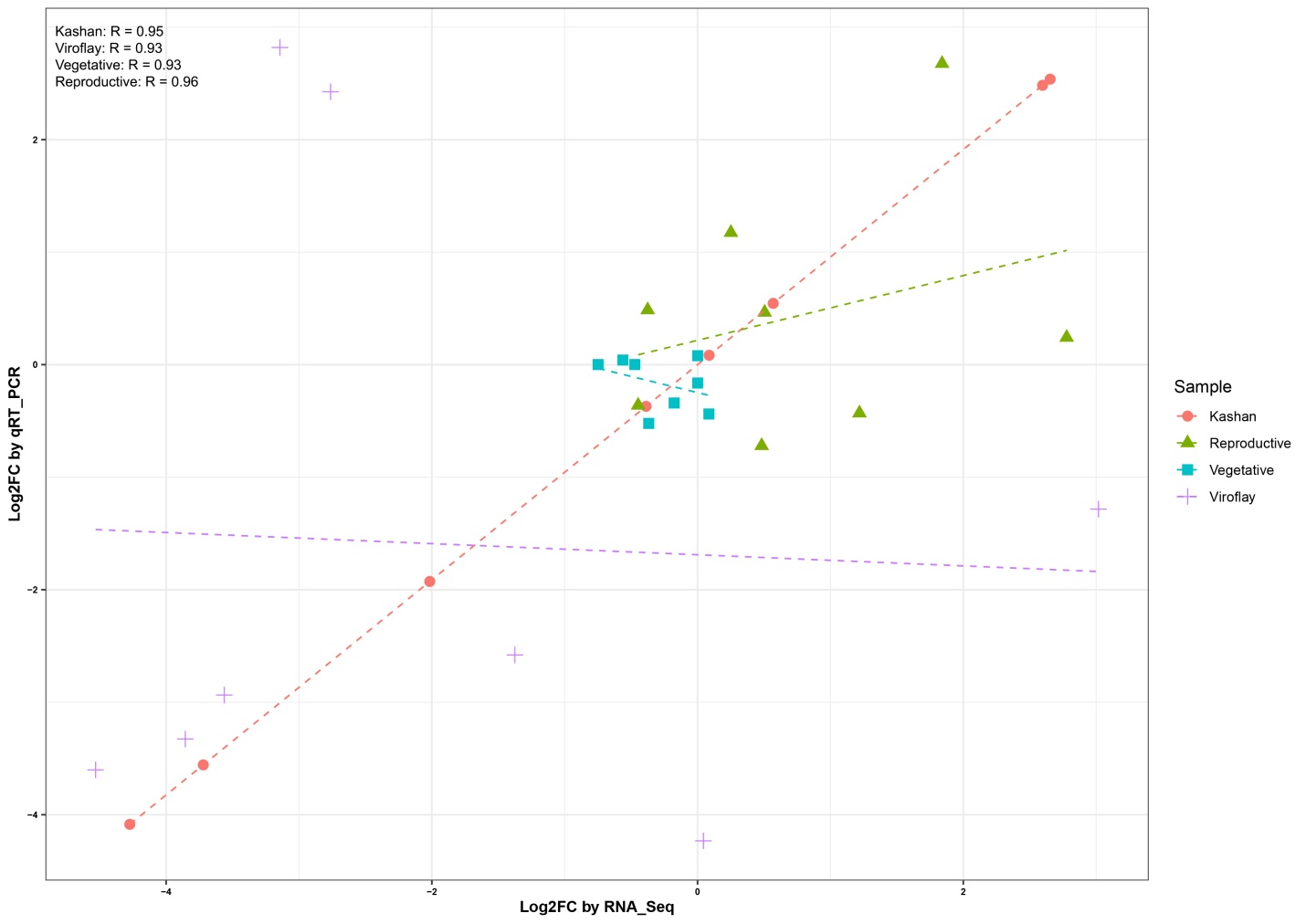


**Figure S5: Gene expression correlation between RT-qPCR and RNA-seq data (Log2 values of the fold change). The Pearson correlation coefficients and linear regression line are indicated.**

**
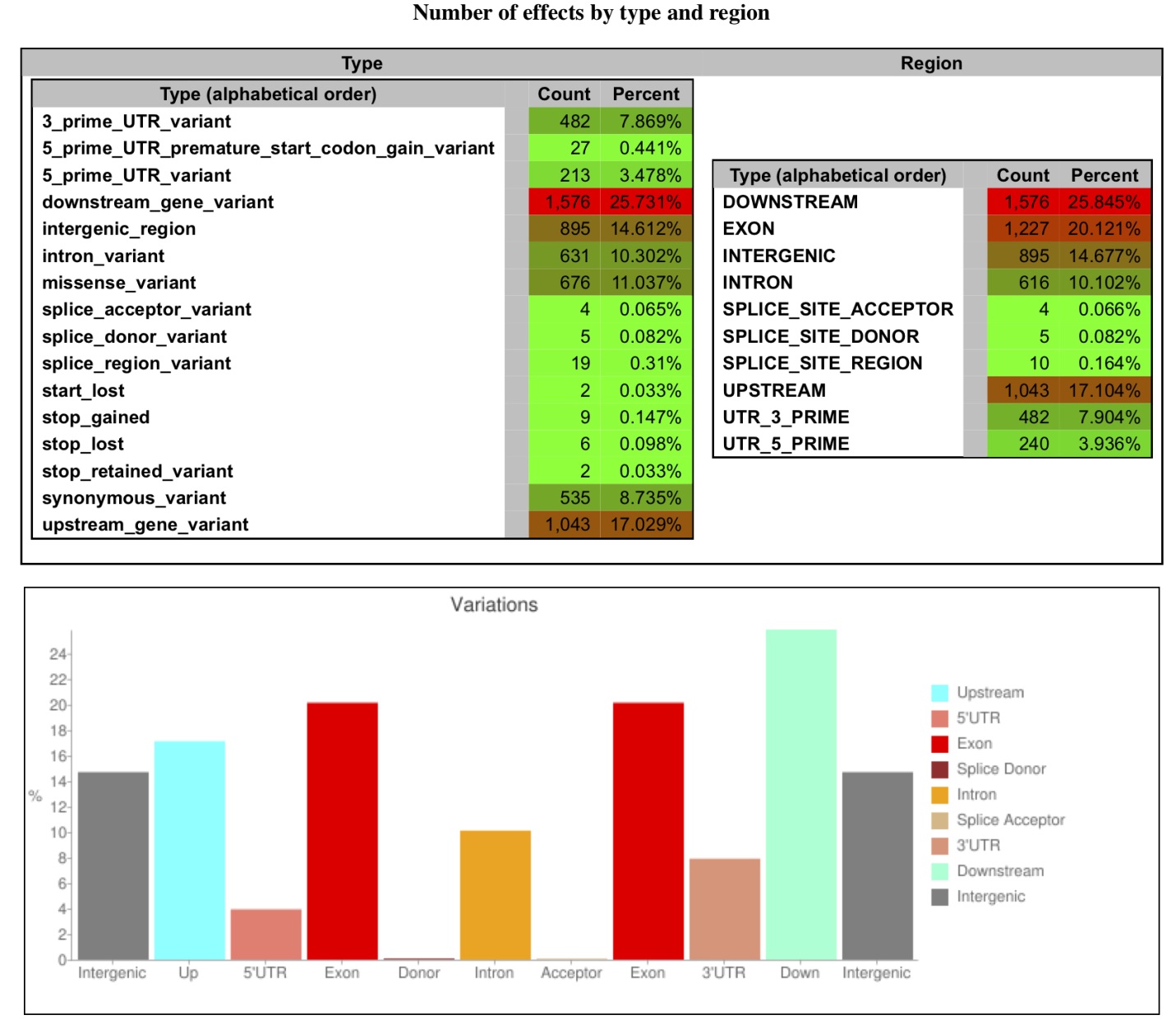
**

**Figure S6: The summary statistics of variant effects by the type and region.**
